# Computational Modeling to Predict Fatigue Behavior of NiTi Stents: What Do We Need?

**DOI:** 10.3390/jfb6020299

**Published:** 2015-05-20

**Authors:** Elena Dordoni, Lorenza Petrini, Wei Wu, Francesco Migliavacca, Gabriele Dubini, Giancarlo Pennati

**Affiliations:** 1 Laboratory of Biological Structure Mechanics, Department of Chemistry, Materials and Chemical Engineering ‘Giulio Natta’, Politecnico di Milano, Milan 20133, Italy; E-Mails: elena.dordoni@hotmail.it (E.D.); wei.wu@polimi.it (W.W.); francesco.migliavacca@polimi.it (F.M.); gabriele.dubini@polimi.it (G.D.); giancarlo.pennati@polimi.it (G.P.); 2 Department of Civil and Environmental Engineering, Politecnico di Milano, Milan 20133, Italy

**Keywords:** peripheral arterial diseases, shape memory alloys, cardiovascular devices, fatigue failure, finite element analyses

## Abstract

NiTi (nickel-titanium) stents are nowadays commonly used for the percutaneous treatment of peripheral arterial disease. However, their effectiveness is still debated in the clinical field. In fact a peculiar cyclic biomechanical environment is created before and after stent implantation, with the risk of device fatigue failure. An accurate study of the device fatigue behavior is of primary importance to ensure a successful stenting procedure. Regulatory authorities recognize the possibility of performing computational analyses instead of experimental tests for the assessment of medical devices. However, confidence in numerical methods is only possible after verification and validation of the models used. For the case of NiTi stents, mechanical properties are strongly dependent on the device dimensions and the whole treatments undergone during manufacturing process. Hence, special attention should be paid to the accuracy of the description of the device geometry and the material properties implementation into the numerical code, as well as to the definition of the fatigue limit. In this paper, a path for setting up an effective numerical model for NiTi stent fatigue assessment is proposed and the results of its application in a specific case study are illustrated.

## 1. Introduction

Nowadays nickel-titanium (NiTi) is commonly used in the production of medical devices. The material capability of undertaking high deformation without residual permanent strain makes NiTi extremely advantageous for endovascular applications.

Referring in particular to the cardiovascular field, NiTi stents are used widely for the treatment of peripheral arterial disease, one of the major manifestations of systemic atherosclerosis [1]. Femoro-popliteal arteries are subjected to a unique set of biomechanical deformations generated by lower limbs daily movements. For example, the superficial femoral artery is exposed to about one million cycles per year of large deformations, including axial compression and extension, bending, and torsion, which are superimposed to the cyclic loading due to the arterial blood pressure (40 million cycles per year) [2]. The introduction of self-expandable stents for the treatment of peripheral arteries has strongly improved the efficacy of the interventional procedure: thanks to pseudo-elasticity, NiTi ensures the recovery from the expanded configuration at the end of each cyclic deformation and hence the preservation of the normal blood stream [3]. However, endovascular treatment success is still undermined by stent long-term fatigue failure due to the cyclic loading, possibly leading to re-occlusion of the artery (in-stent restenosis) [4,5]. Hence, the assessment of the risk of stent fatigue rupture is of primary importance to ensure the effectiveness of stenting procedure. At present, this is commonly performed through *in vitro* tests. The experimental fatigue tests, usually regulated by international standards (e.g., ISO 25539 [6], FDA Guidance [7] and ISO 5840 [8]), provide an immediate assessment of durability of the device subjected to a particular cyclic loading condition, for a given number of cycles. However, they still suffer from a number of disadvantages.

- High costs and long duration. A great number of devices must be tested for millions of cycles in order to ensure statistically significant results. Moreover, experimental campaigns often require expensive instruments to setup external loadings, boundary conditions and measurements of the quantities of interest.
- Difficulty in reproducing the *in vivo* environment. Physical reality has a high complexity, in terms of anatomical features, material properties of biological tissue and cyclic loading conditions, which is known to change from patient to patient. For those reasons, experimental tests are usually simplified and poorly representative of the real conditions.
- Difficulty in assessment the biomechanical quantities. Measurement is always a critical point in experimental tests due to challenging calibration of the instruments and measurement errors (bias, sensor resolution, and accuracy). Moreover, fatigue tests give only the final result (safety or failure), often missing the exact number of cycles and any information on the stress state in the device.

During the last decades, numerical simulations, in particular by means of the finite element (FE) method, have become a well-recognized and widely adopted tool to investigate biomechanical issues. This is mainly due to their advantages in terms of reduced costs, high flexibility and ability of assessing important biomechanical quantities. The creation of virtual geometrical models of the real biological environment (by means of CAD software or image-based reconstructions from medical images) allows one to easily and quickly modify some parameters, for instance geometrical features, boundary conditions and external loadings. Moreover, numerical models provide direct and continuous access to many quantities (such as stress/strain field through the device) during the whole test simulation.

Regulatory authorities acknowledge the possibility of performing FE analyses instead of experimental tests for the assessment of medical devices. However, the quality of the numerical results is not automatically ensured and great caution must be taken during the implementation of such models as well as during the interpretation of the outputs. Beside the aforementioned benefits, confidence in computational methods is only possible after the verification of the mathematical foundation of the model and the validation of the results against experimental data. In particular, when the aim of simulations is the assessment of NiTi device fatigue resistance, a special attention should be paid to the correctness of the device geometry description and of the material properties implementation into the FE code, as well as to the material fatigue limit. Indeed, NiTi elastic and fatigue properties are strongly dependent on the device dimensions and on the whole treatments (thermal and surface finishing) undergone by the device during the manufacturing process.

In this paper we propose a path, combining experimental and numerical tests, which can be followed to set up an effective FE model of a NiTi stent suitable for fatigue assessment. We focus on those steps that precede the proper device fatigue FE analyses, but are fundamental for getting reliable results from them. These steps are: (i) development of the FE stent model; (ii) set-up of experimental tests for material mechanical parameter identification and preliminary validation of the stent model; (iii) characterization of the material fatigue behavior; and (iv) definition of the fatigue criterion and validation. In the following Section, the proposed path is described in detail, while in the “Results” Section, it is applied to a specific commercial stent.

For the numerical simulations the commercial FE code ANSYS Mechanical APDL (Ansys Inc., Canonsburg, PA, USA) was used.

## 2. Materials and Methods 

### 2.1. Development of NiTi Stent FE Model

The accuracy in the reconstruction of the stent geometry is one of the basic points for a good fatigue numerical prediction. NiTi stents are manufactured in a fully-expanded configuration, with a diameter larger than that of the target anatomical site (*i.e.*, oversizing). Therefore, they are compressed through the crimping procedure and inserted into a retractable sheath. As a consequence, the FE analyses should reproduce the whole implantation procedure, without neglecting the residual stress and strain fields induced by the crimping phase. Accordingly, the device model could be built in two different ways, depending on the acquired data. The former approach consists of obtaining directly geometrical data from the device in the fully expanded configuration. Precise measurements of V-strut thickness, width, length and opening angle can be taken from optical images using stereo microscopy [9] or from microcomputer tomography [10,11]. The advantage of this procedure is that the fully expanded configuration is immediately obtained, but errors can be present due to inaccuracy in measures and variability related to the single device.

The latter approach requires that the drawings of the laser cut shape are available. In this case, the geometry is simply obtained by extruding the 2D planar sketch for the laser machine input, to a length equal to the NiTi tube thickness and then wrapping it by 360°. Although the initial dimensions are now exact, the 3D model still needs to be expanded. The annealing process during manufacturing also needs to be taken into account, in order to achieve the devices expanded configuration without residual stresses. These simulation steps can be additional sources of error.

In both cases, the geometrical model has to be discretized in finite elements. This is an important step because mesh density can strongly influence the analysis results. A high-quality mesh is obtained firstly by partitioning the stent volume, dividing the curved and the straight regions, thus facilitating its regular discretization; and secondly, subdividing the device cross-section by means of a 2D quadrilateral regular mesh and dragging the mesh along the strut surface to create 3D solid elements. Due to stress concentrations and high stress gradients in the curved portions, they are usually more finely discretized, while a coarser mesh to decrease the computational cost of the analyses characterizes the straight regions. The optimal mesh, corresponding to the best compromise between accuracy in the results and computational costs, can be selected through a sensitivity analysis.

### 2.2. Specimens for NiTi Mechanical Parameter Identification

Experimental characterization of NiTi mechanical properties is a delicate problem. Indeed, the small dimensions of the stent struts (few hundreds of microns) as well as the whole procedure for producing the stent may strongly influence the NiTi performance. Heat treatments modify the mechanical properties and chemical etching/electropolishing change the surface and hence the fatigue behavior. Accordingly, particular attention has to be devoted to the definition of the specimen geometry that must satisfy different requirements.

- Reproducing the size of the stents struts, since the size effect is relevant in determining the fatigue properties.
- Having a gauge length in which a uniform deformation occurs; in this way stress and strain can be calculated analytically or by means of a simple FE analysis.
- Allowing a high number of fatigue results for each experimental condition to perform a statistical analysis.

**Figure 1:**
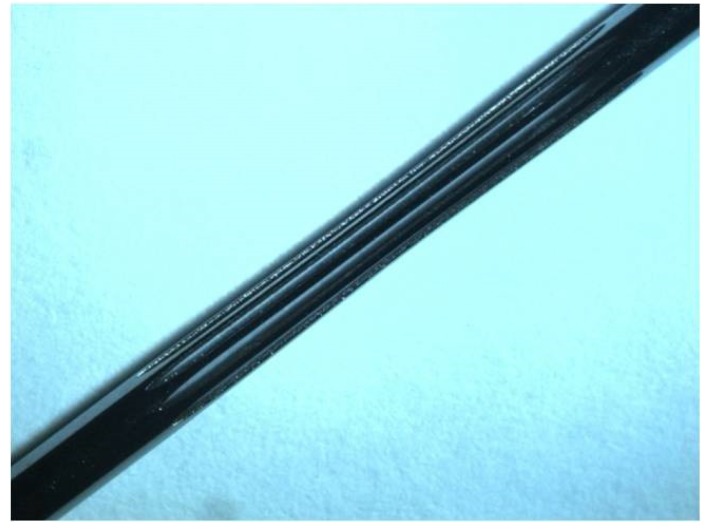
Specimen designed for mechanical tests.

After preliminary calculations, a tubular specimen was selected as the final design for axial tensile or positive sinusoidal (non-zero mean strain) deformation, with wires cut in parallel along the circumference, and exhibiting the typical “dog bone” shape of specimens for tensile tests (Figure 1). The tubular ends allow a precise axial positioning into the grips of the testing machine. The cross-section dimensions, gauge length and number of wires along the circumference can be chosen by considering the stent strut dimensions and limits related to the full scale force range of the testing machine. The dimensions of the fillet zone between each wire have to be selected to ensure that the whole force applied to the specimen ends is transferred on its gauge length.

### 2.3. Static Tests for Material Characterization and Validation

**Figure 2:**
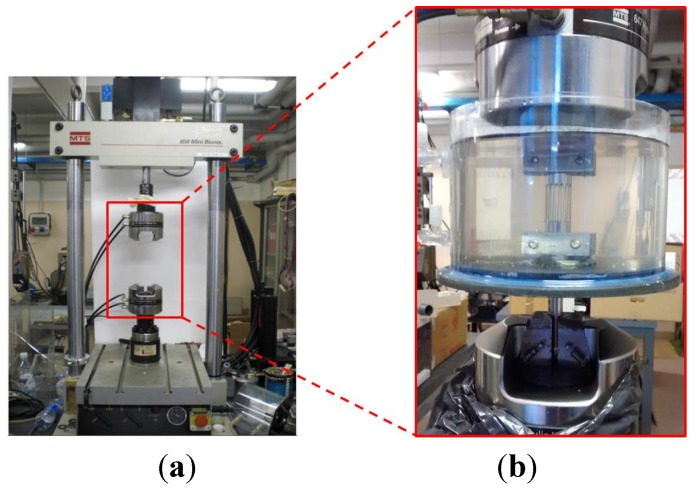
The closed-loop testing machine used for mechanical tests (**a**) with a particular of the temperature control system (**b**).

The use of NiTi constitutive models in numerical simulations requires the identification of material properties expressed through a set of parameters. Hence different experimental tests have to be performed. In the case of the NiTi model implemented in ANSYS [12], simple static tensile tests are sufficient. In this work, they were performed using a closed-loop, servo-hydraulic, testing machine (Figure 2a) according to the methodology prescribed in the standard ASTM F 2516-07 [13] (tension loading up to 6% of strain, unloading up to initial configuration and again tension loading up to fracture).

Because of the NiTi temperature-depending mechanical behavior, a temperature control system was used. It consisted of a hydraulic system composed of a roller pump, an upstream reservoir controlling water temperature, a Plexiglass^®^ chamber connected to the testing machine and provided with a terminal jaw that grips the specimen, as well as connecting pipes among each component (Figure 2b).

Once the material parameters for the computational analyses are identified, a validation of the constitutive relationship should be conducted. An effective way is by comparing numerical and experimental results of a crimping test.

In this work, three cycles of crimping and release in a temperature-controlled environment (37 °C, through hot air flux), from stent fully expanded configuration up to a defined diameter, were accomplished using radial expansion force testing equipment (displacement resolution 0.01 mm; force transducer 11.25/22.5 N). The output measurement was the radial force. In the computational analyses, the crimping was simulated by means of a cylindrical rigid surface, placed along the axis of the stent and then contracted in the radial direction, imposing a negative radial displacement on its nodes, while keeping fixed axial and circumferential directions. A rigid surface-to-surface contact was created between the two surfaces, with a normal stiffness set equal to 0.01 and a low value of friction, equal to 0.05.

It is worth noting that it is sometimes difficult to have the NiTi stent specimens as previously described. One possible way to overcome this limitation, provided samples of the final device are available, is to perform simple experimental tests (such as axial tensile tests) on the device, to identify the material parameters and then perform separate additional tests (such as crimping tests) to validate the model. This approach was followed to study the Zilver™ peripheral stent from Cook Medical. The results of a static tensile (80%) and compression (20%) axial test were used to calibrate the material parameters starting from literature values. Then a crimping test was performed for validation.

### 2.4. Cyclic Tests for Material Fatigue Characterization

The fatigue characterization of NiTi is of primary importance for the design assessment of cardiovascular devices that experience millions of *in vivo* loading cycles. Moreover, the strong non-linearity of the material pseudo-elastic stress/strain curve, characterized by two different plateau regions, requires that the whole deformation history experienced by the device and a strain-based approach should be taken into account in designing the testing protocol.

**Figure 3:**
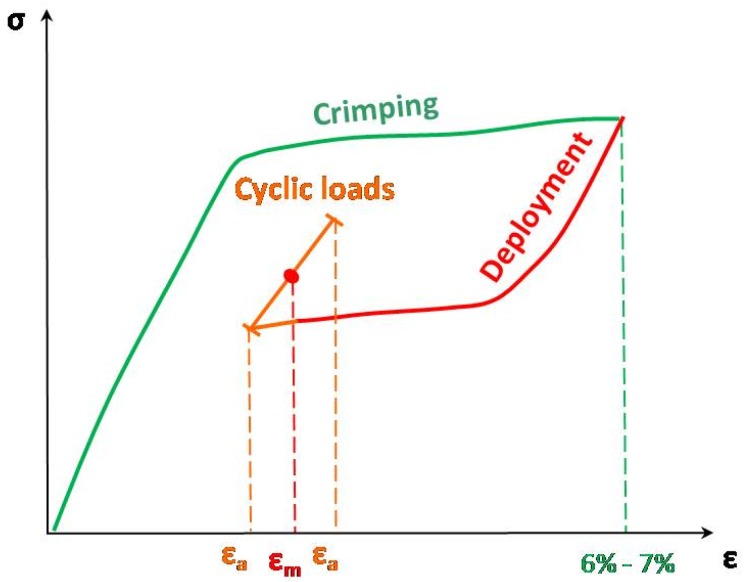
Sketch of the fatigue testing condition adopted, reproducing the strain history that NiTi is subjected during stenting.

During the stenting procedure, the crimping and the following self-expansion produce mainly bending deformations, having their maximum at the apex of the V strut of each ring. Assuming that tensile strain is the most dangerous solicitation for fatigue behavior and that the material is free from residual strain at the end of the production phase, the most critical points during crimping are subjected to tension and they partially recover the initial configuration because of the self-expansion during deployment. As shown in Figure 3, during the crimping and subsequent self-expansion phases, each stent point ideally describes a σ/ε curve up to an unloading strain value indicated as ε_m_. The cyclic forces, following the deployment, superimpose an alternate strain (ε_a_) to ε_m_ and induce small, mainly elastic loops. Accordingly, the following testing protocol for fatigue tensile tests on NiTi specimens was defined: (i) tensile force at a velocity of 0.8 mm/min up to 6%–7% strain, to replicate the stress-strain state experienced by devices during the crimping process; (ii) unloading at 0.8 mm/min up to a predefined value of mean strain, representative of device deployment inside an artery. Values in the range 1%–6% were chosen, according to different stent oversizing; and (iii) cyclic forces, at 50 Hz frequency, with different amplitude strains defined according to literature data [14]. The tests were conducted undern displacement control, in a temperature-controlled environment, using the same set-up described for the static tensile tests.

The “testing for survival” approach was adopted, up to a maximum number of cycles equal to 10^7^ cycles, corresponding to 10 years of usage. The material fatigue strain limit for 10^7^ cycles was obtained plotting the results of fatigue tests in the constant life diagram (mean strain *versus* strain amplitude) and distinguishing among fractured and survived specimens.

**Figure 4:**
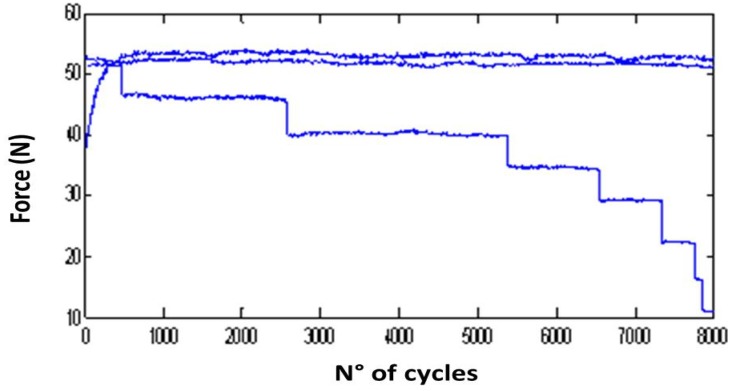
Example of detection of mean axial force drops at different number of cycles, corresponding to fracture of wires. Each line represents one loop of acquired data, made at 8000 cycles.

During the fatigue tests, a peak-to-valley acquisition was used: for each loading cycle, the maximum and minimum values of the axial force were acquired and stored in a data file in order to evaluate its trend for the whole testing duration as a function of number of cycles. Since a wire fracture determines a decrease in the specimen resistant section, the force signal acquired shows an evident drop, greater than the minimum force resolution of the testing machine. Therefore, this method allowed the detection of the exact number of cycles for each wire fracture (Figure 4). This information is useful for defining material limit curves for a number of cycles lower than 10^7^.

### 2.5. NiTi Fatigue Criterion

Cyclic loadings (axial force, bending or torsion) cause multi-axial strain states in peripheral stents. Accordingly, for the fatigue assessment of these devices, it is necessary to define a fatigue criterion to compare the multi-axial strain state with the material fatigue limit, obtained by uniaxial tests. The common approach followed by several authors [15,16,17,18,19], and also proposed within this work, is to express the NiTi fatigue criterion in terms of the first principal value of the mean and amplitude strain ($\varepsilon_{m}^{I}$, $\varepsilon_{a}^{I}$), assuming the tensile strain as the most critical condition for fatigue failure. A procedure was implemented in the FE code to get these data when stent fatigue simulations are performed. It consists of: (i) extracting the strain tensors at the maximum and minimum values of the cyclic force, at each Gauss point; (ii) calculating mean and alternate strain tensors using an APDL script; and (iii) obtaining the first principal strains for mean and amplitude strain tensors by using classical eigenvalue analysis.

The choice of the fatigue criterion is a fundamental step in the stent fatigue assessment procedure. Hence, a validation of the criterion would be desirable. To accomplish this, (i) perform some experimental tests on real devices, that are easily reproducible by numerical simulations and are able to give different results (fracture/safety); (ii) simulate the same tests with the FE code and compare the first principal strains for each Gauss point with the limit fatigue curve; and (iii) verify that simulations predict fracture for the same loading cases and in the same positions of experimental tests.

Indeed, a fatigue criterion based on principal strain values is suitable for stents having classical design with V-shaped rings connected by links and subjected to axial and bending forces. In this case, stent struts mainly work in bending and compression/tension strains (diagonal terms in the strain tensor) are predominant. However, medical devices may be subjected to more complex modes of loading in the body (a combination of axial and radial compression, bending and torsion) and may have different designs such that all strain tensor components have comparable values. Under these conditions, the principal strain criterion may be too simplistic and hence not accurate enough. In the literature, some authors suggest using different criteria, such as Von Mises criterion for ductile materials [20] or Smith-Watson-Topper criterion [21], which take into account the influence on fatigue life prediction of all strain tensor components and of stress, respectively. Lastly, it is the opinion of these authors that the principal strain criterion may be used with proper attention paid, however a study comparing different fatigue criteria may be useful to improve NiTi stent assessment. 

## 3. Results 

In the following, the results obtained applying the described procedure to the case of the Maris Plus™ peripheral stent from Medtronic Endovascular Therapies are summarized. For confidentiality reasons, some data are not reported; however, this does not invalidate the efficacy of the reported examples.

### 3.1. Development of NiTi Stent FE Model

**Figure 5:**
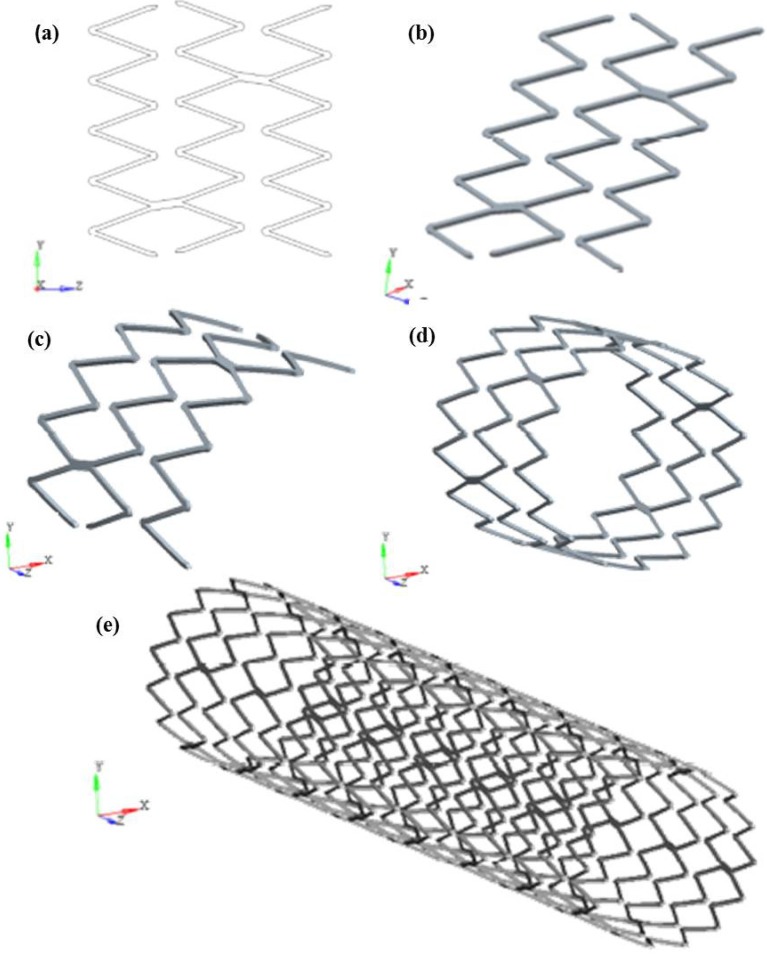
Development of the geometric model for finite element (FE) analyses of a commercial stent: (**a**) the 2D sketch of the repetitive unit is first (**b**) extruded in the radial direction and then (**c**) rotated by 120° to build the 3D geometry of the stent repetitive unit; then the unit is copied in (**d**) circumferential and (**e**) axial directions, obtaining the whole stent.

To create the geometrical model of Maris Plus™ stent, the CAD software ProENGINEER Wildfire 4.0™ was used, after the acquisition of physical model dimensions. In order to fasten the creation of the model, the symmetries of the stents were acknowledged and a repetitive unit was identified, consisting of one third of three V-strut crowns. The starting point was the 2D sketch of the repetitive unit (Figure 5a): it was first extruded in the radial direction with an offset equal to the stent thickness (0.2 mm) (Figure 5b), and then rotated by 120° in order to achieve the 3D geometry of the stent repetitive unit (Figure 5c). Once we obtained the repetitive unit, the whole stent was generated by replicating the desired number of such units in the circumferential (Figure 5d) and axial directions (Figure 5e).

**Figure 6:**
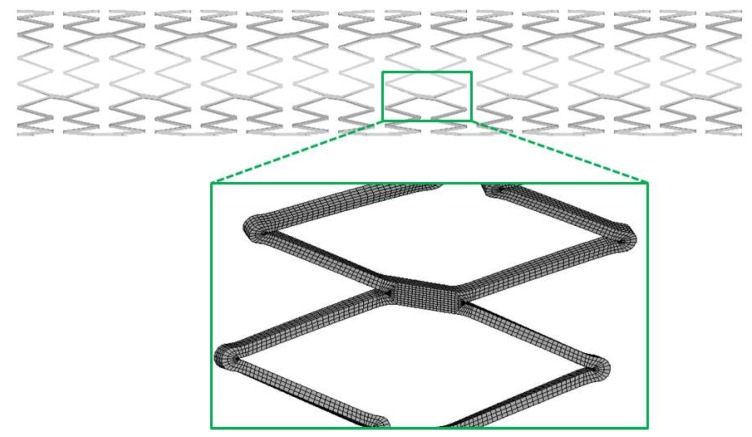
Detail of the stent model mesh.

The mesh used in this particular case consisted of three elements in the width and four elements in the thickness of the stent cross-section and resulted in a total amount of 277,000 solid elements with eight nodes and full integration algorithm (Figure 6).

### 3.2. Specimen for NiTi Mechanical Parameter Identification

**Figure 7:**
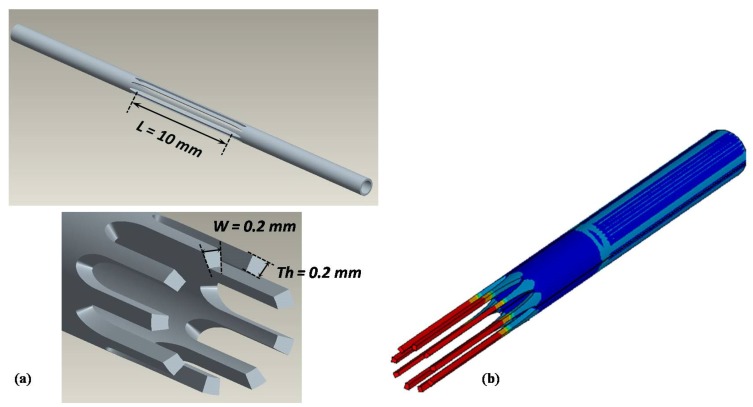
(**a**) CAD model of NiTi specimen for static and fatigue characterization. The significant dimension of gauge length (*L*), wires width (*W*) and thickness (*Th*) are reported. (**b**) Symmetrical FE model of a tensile test up to 5%: the total maximum strain, uniform and distributed along each wire, is equal to 4.459%, which means that almost the whole load applied to the specimen is transferred on its gauge length.

A specimen having nine wires along the circumference with cross-section of 0.2 mm × 0.2 mm and gauge length equal to 10 mm was designed (Figure 7a), considering that the load full-scale range of the testing machine was 1500 N.

The capability of the specimen to ensure that the whole deformation applied to the specimen ends is almost totally transferred to its gauge length was assessed by FE analyses. Results (Figure 7b) show that the maximum strain is uniform and localized in the gauge length once an axial load is applied.

Specimens with the described design were laser cut from the same tubes and according the same procedure used for stent production.

### 3.3. Static Tests for Material Characterization and Validation

**Figure 8:**
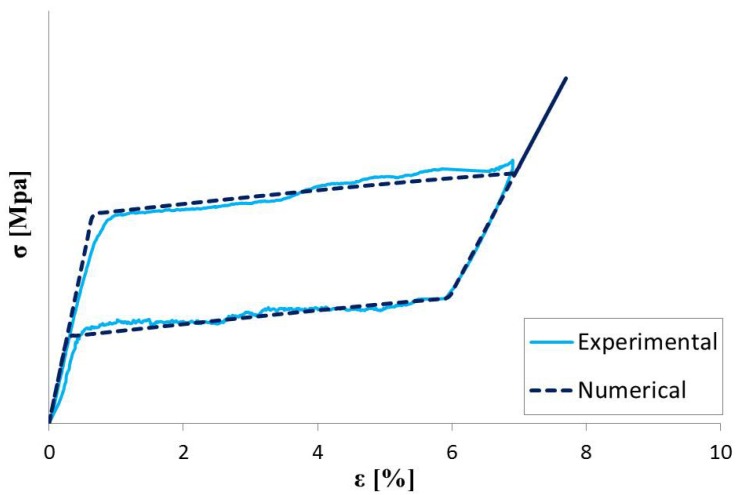
Experimental and numerical stress-strain curve for NiTi specimens.

Tensile tests at controlled temperature (37 ± 2 °C), corresponding to the *in vivo* condition, were performed. Thanks to the designed specimen shape, it was easy to convert the force/displacement result into a σ/ε relationship and calculate the material parameters for ANSYS constitutive model (Figure 8).

**Figure 9:**
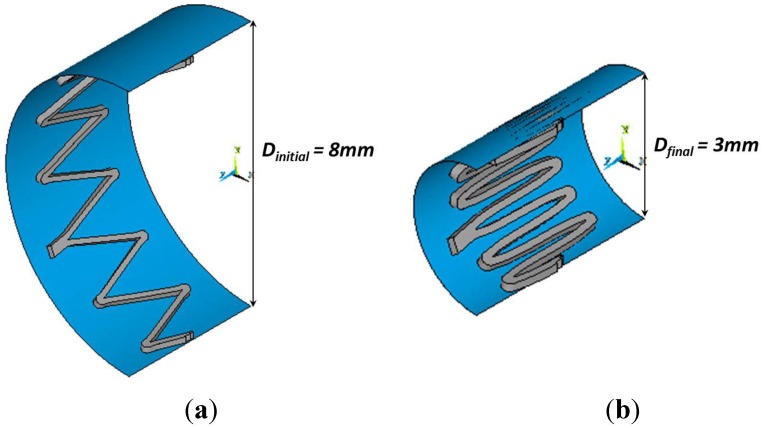
Numerical (half) model of the simplified crimping simulation on one ring of peripheral stent. This is performed through a rigid contact, defined between the stent outer surface and the external cylinder one: starting from the fully-expanded configuration (**a**), the cylindrical rigid surface moves in negative radial direction until the stent reaches its final crimped shape (**b**).

To validate the numerical model, crimping tests and simulations were performed as described in Section 2.3. In particular, four specimens composed of eight rings each were tested, starting from a fully expanded diameter of 8 mm up to a diameter of 3 mm. In the FE analysis only a repetitive unit was considered, taking advantage of the Maris Plus™ repetitive axial pattern (Figure 9). The whole hoop force was then calculated by multiplying the numerical force obtained on the reduced model for a number of times (8) allowing the reproduction of the whole device tested.

**Figure 10:**
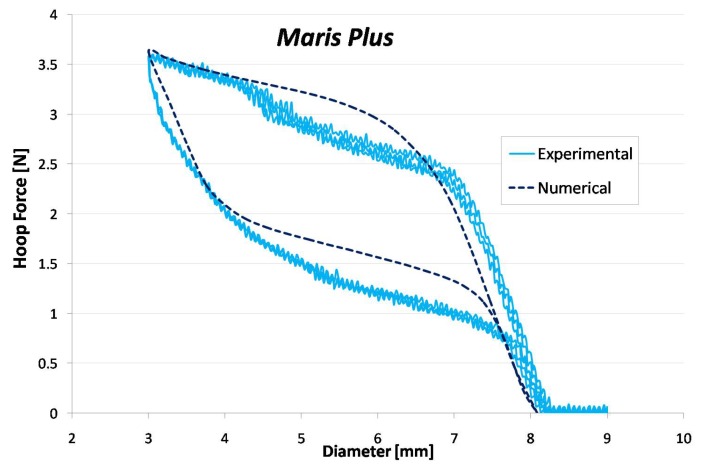
Comparison between experimental and numerical hoop force-diameter curves for the stent. The reported experimental curve represents the mean curve between all the performed tests (4).

A good agreement can be noticed between experimental and computational results of crimping test in terms of hoop force (Figure 10), with small differences that might be related to friction effects.

**Figure 11:**
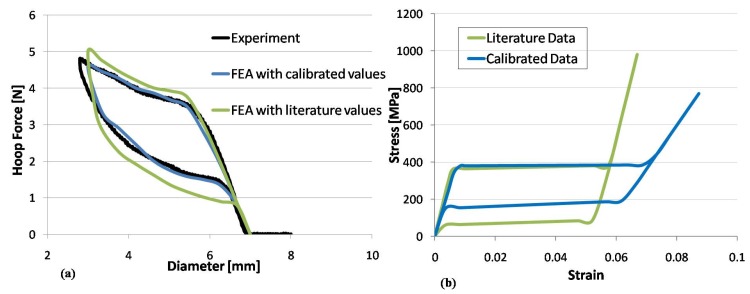
(**a**) Comparison between experimental and numerical hoop force-diameter curves for Zilver™ peripheral stent. In the finite element analyses, material parameters were set using literature data (green curve) and calibrated data (blue line) obtained from an identification procedure based on experimental tests on the whole device. (**b**) Plot of the numerical stress-strain curves obtained from literature data (green curve) and calibrated data (blue line).

The effectiveness of the identification procedure proposed in Section 2.3, for cases where *ad hoc* specimens cannot be produced, is evident in Figure 11a where the experimental hoop force-radial displacement curve of Zilver™ stent (black line) is compared with numerical results obtained using calibrated parameters (blue line) and literature data (green line) for the material constitutive model. The comparison of the corresponding stress-strain curves (Figure 11b) highlights the necessity of performing material characterization for each stent type.

### 3.4. Cyclic Tests for Material Fatigue Characterization

**Figure 12:**
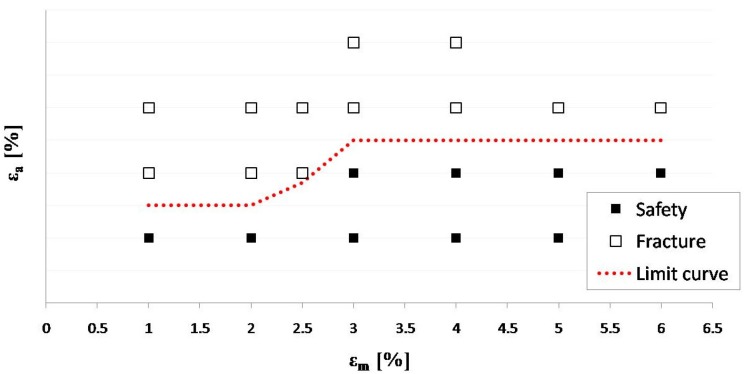
Constant life diagram for NiTi specimens for *N* = 10^7^.

In the following, the results of fatigue characterization for NiTi specimens is described. The protocol was applied to 189 wires (corresponding to 21 specimens) that were tested cyclically at various combinations of mean and amplitude strain up to 10^7^ cycles. The results were plotted as mean strain *versus* strain amplitude as a constant life diagram (Figure 12). In the diagram, the specimens that survived 10^7^ cycles are shown as solid black squares, whereas those specimens that fractured are shown as open squares; the red dotted line between failure and safety points represents the fatigue strain limit for the material. The limit curve trend is coherent with literature data [14], showing two horizontal plateaus for mean strains ranging between 1%–2% and 3%–6%, and a rising line between 2% and 3% of mean strain. The results confirm the improvement of the NiTi fatigue limit for greater mean strain values, while the plateau values in terms of amplitude strain were found to be strongly dependent on surface finishing and manufacturing processes.

Some authors [16,22] suggest simplifying the fatigue limit curve with a constant line corresponding to the lower value. This approach makes the fatigue analyses easier, but it is too conservative and may lead to an incorrect interpretation of the stent *in vivo* behavior. Indeed, mean amplitude mainly depends on oversizing, thus, the fatigue risk for the stent is greater where the oversizing is less, *i.e.*, where plaque has a small thickness or is even absent [23].

### 3.5. NiTi Fatigue Criterion

The NiTi fatigue criterion, based on mean and amplitude first principal strains, was selected in view of FEA for fatigue assessment. The suitability of this choice was verified applying the procedure proposed in Section 2.5 to Maris Plus™ stents. The same set-up of static tests for material characterization (Section 2.3) was adopted. Experimental cyclic axial tests on stents in their fully expanded configuration were performed considering two different lengths for the specimens, equal to eight rings (*L* = 22.6 mm) and four rings (*L* = 11.3 mm), respectively. Several simple axial fatigue tests in displacement control and temperature-controlled environment (37 ± 2 °C) were conducted, each time changing the imposed mean and alternate displacement, in order to obtain safe and unsafe conditions. The testing procedure consisted of an initial elongation up to 80%, unloading up to a defined displacement, and cyclic loading phase at a frequency of 20 Hz. The testing conditions in terms of mean and alternate displacement values (*u*_m_, *u*_a_) and final results (fracture or survival of the stent after 10^7^ cycles) are listed in Table 1, for both eight-ring and four-ring stents.

**Table 1:** Cyclic axial tests on peripheral stents: testing conditions in terms of mean and alternate displacement values (*u*_m_, *u*_a_) expressed in mm and final results after 10^7^ cycles.

Tests	8 Rings			4 Rings		
	*u*_m_	*u*_a_	Fracture	*u*_m_	*u*_a_	Fracture
Test 1	11.05	±2.55	Yes	5.525	±1.275	Yes
Test 2	8.4	±2.2	Yes	4.2	±1.1	Yes
Test 3	8.55	±2.55	Yes	4.275	±1.275	Yes
Test 4	11.5	±2	No	5.75	±1	Yes
Test 5	−2.39	±2.15	Yes	6	±1.28	Yes
Test 6	–	–	–	4	±0.25	No

**Figure 13:**
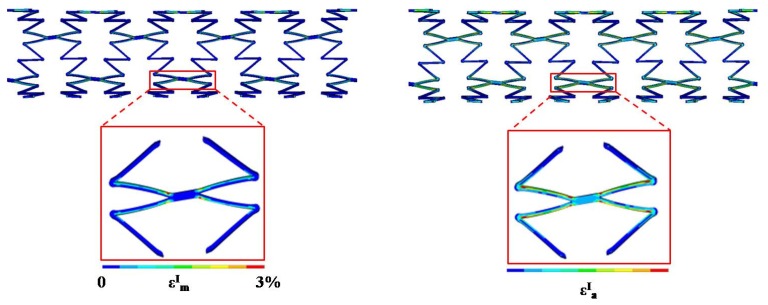
Contour map of mean and amplitude strain values due to the cyclic loading condition defined for test 1 on an eight-ring stent; in the red boxes, magnifications are shown of the most stressed areas.

FE simulations reproducing the same experimental conditions were performed. The previously described numerical stent model (geometry and material parameters) was used. Displacement boundary conditions were applied by means of Multi-points Constraints Elements (MPC184), connecting an external master node to the nodes of each stent end; displacements in axial direction were imposed to one master node, while the other was kept fixed. Each FE analysis allowed us to determine the highly stressed zone of the stent in terms of mean and amplitude values of the first principal strain. For example, the mean and amplitude strain distributions for test 1 condition and eight-ring stent are shown in Figure 13: the most stressed zones are always located in two areas, identified as the link and the V-strut directly connected to the link.

**Figure 14:**
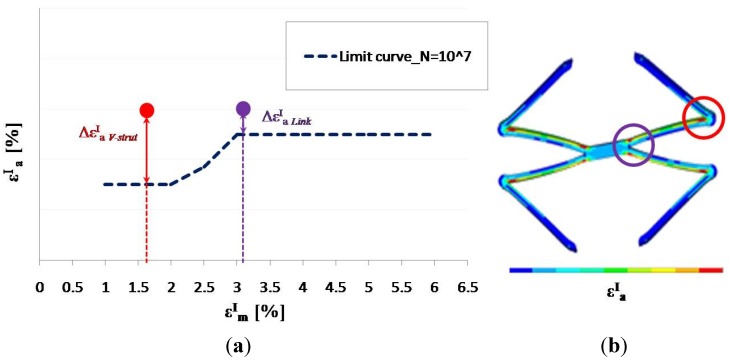
Test 1 condition on eight-ring stent: mean and amplitude strain values in the link and in the V-strut are plotted on the constant-life diagram. Red and violet dots (**a**) include pairs of mean and amplitude values of all the finite elements belonging to the two most stressed zones (**b**).

For each FE analysis, the values ($\varepsilon_{m}^{I}$, $\varepsilon_{a}^{I}$), related to the elements belonging to the two highly stressed zones were plotted on the constant-life diagram and compared with the material fatigue limit at 10^7^ cycles in order to find the most critical point for fatigue failure in terms of distance from the limit curve. Figure 14 shows how the criterion indicates the V-strut as the most critical area for fatigue failure in test 1 condition for eight-ring stent. Even if the amplitude strain in the two highly stressed zones (red and violet dots) is the same, the distance from the material fatigue limit is greater for the V-strut, due to its low mean strain value.

**Figure 15:**
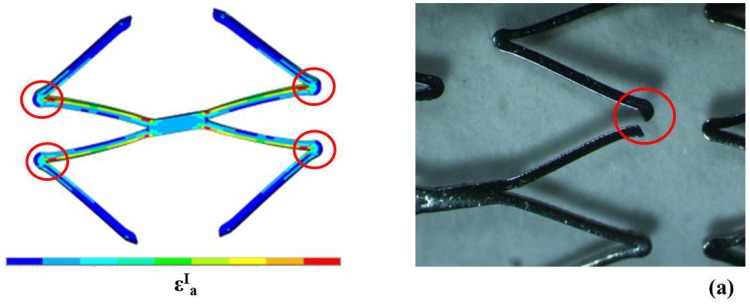

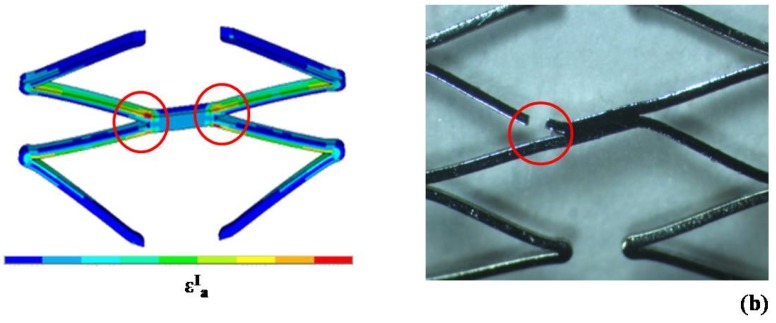
Agreement between numerical and experimental results of preliminary tests on stents for NiTi fatigue criterion validation. Red circles indicate the most critical areas for fatigue fracture predicted by the numerical model that coincide with the experimental fracture point.

**Figure 16:**
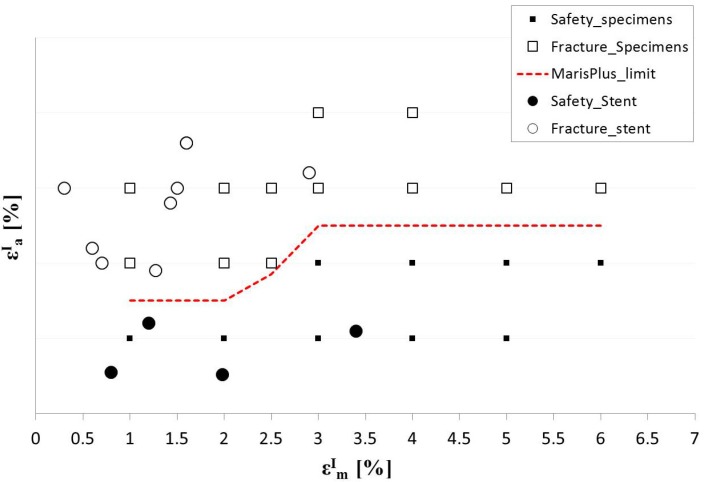
Constant-life diagram from fatigue tests on NiTi dog bone specimens and cyclic axial tests on peripheral stents for different conditions of mean and amplitude strain.

The comparison between computational and experimental results showed the good ability of the finite element model to predict the fatigue behavior of all the tested devices, also locating fracture position when fractures occurred (Figure 15). Moreover, a perfect overlay was obtained for the experimental stent axial fatigue-life data on the dog bone fatigue-life data (Figure 16). These findings allow the validation of the fatigue resistance criterion adopted for Maris Plus™ stent, at least in the case of simple loading conditions.

## 4. Conclusions 

The complexity of cardiovascular device applications, together with the complexity of NiTi behavior, requires particular attention in the assessment of the fatigue fracture risk of NiTi stents. Numerical simulations are a valuable support for the fatigue analysis of NiTi cardiovascular devices and provide quantitative information about the stress/strain field caused by an imposed load, which could hardly be investigated with analytical or experimental methods.

In order to make reliable numerical predictions from fatigue analyses, it is fundamental to represent the device under study as accurately as possible, in terms of geometry and material properties, both from a static and a cyclic point of view. In this paper, we suggest and describe in detail a possible path to obtain an accurate and validated stent computational model suitable for FEA fatigue assessment. For the construction of the CAD model, it is necessary that real devices are available (and can be easily measured) or post-laser cutting drawings are accessible. The material properties knowledge is a non-trivial issue to solve. The most efficient way to get a more thorough understanding of NiTi mechanical behavior is to perform an accurate experimental campaign of material characterization on dog-bone specimens, designed according to proper shape and dimensional constraints, and subjected to the same manufacturing treatments of the final device. In this paper, an example is described for an *ad hoc* developed specimen, together with its use in static and fatigue tests for material characterization. Moreover, an alternative procedure is proposed to obtain the mechanical parameters directly from devices in the absence of specific specimens. Once the material parameters for the computational analyses are identified, we suggest validating the constitutive relationship by comparing numerical and experimental results of a crimping test.

The selection of the material fatigue criterion is another crucial point and its validation is fundamental. Our proposal is to perform a number of simple axial fatigue tests on stents in their fully expanded configuration, easy to reproduce by numerical simulations and able to give different results (fracture/safety).

Once all these preliminary steps have been performed, the stent model is ready to be used in FEA fatigue analyses. Clearly, appropriate boundary conditions, loads and other model parts (e.g., tubes, vessels, crimping surfaces) will be added according to the situations (*in vivo* or *in vitro*) that have to be simulated.
